# PC-3-Derived Exosomes Inhibit Osteoclast Differentiation by Downregulating miR-214 and Blocking NF-*κ*B Signaling Pathway

**DOI:** 10.1155/2019/8650846

**Published:** 2019-04-01

**Authors:** Yang Duan, Zhiwen Tan, Minsheng Yang, Jianjun Li, Chun Liu, Chengqiang Wang, Fu Zhang, Yanglei Jin, Yihan Wang, Lixin Zhu

**Affiliations:** Department of Spinal Surgery, Zhujiang Hospital, Southern Medical University, Guangzhou, China

## Abstract

Prostate cancer is a serious disease that can invade bone tissues. These bone metastases can greatly decrease a patient's quality of life, pose a financial burden, and even result in death. In recent years, tumor cell-secreted microvesicles have been identified and proposed to be a key factor in cell interaction. However, the impact of cancer-derived exosomes on bone cells remains unclear. Herein, we isolated exosomes from prostate cancer cell line PC-3 and investigated their effects on human osteoclast differentiation by tartrate-resistant acid phosphatase (TRAP) staining. The potential mechanism was evaluated by qRT-PCR, western blotting, and microRNA transfection experiments. The results showed that PC-3-derived exosomes dramatically inhibited osteoclast differentiation. Marker genes of mature osteoclasts, including CTSK, NFATc1, ACP5, and miR-214, were all downregulated in the presence of PC-3 exosomes. Furthermore, transfection experiments showed that miR-214 downregulation severely impaired osteoclast differentiation, whereas overexpression of miR-214 promoted differentiation. Furthermore, we demonstrated that PC-3-derived exosomes block the NF-*κ*B signaling pathway. Our study suggested that PC-3-derived exosomes inhibit osteoclast differentiation by downregulating miR-214 and blocking the NF-*κ*B signaling pathway. Therefore, elevating miR-214 levels in the bone metastatic site may attenuate the invasion of prostate cancer.

## 1. Introduction

Prostate cancer is one of the most common malignant tumors, with bone as the preferential metastatic site [1, 2]. Without effective intervention, persistent invasion of prostate cancer will soon lower the quality of life of affected patients and even result in death [3]. Bone metastatic lesions can be divided into two categories, osteoblastic or osteolytic, depending on the radiographic characteristics. The differences are caused by an imbalance between bone formation and bone resorption, i.e., whether osteoblasts or osteoclasts are dominant [4, 5]. Prostate cancer usually leads to osteoblastic bone metastasis. Studies have found that prostate cancer cells release many cytokines to promote osteoblast differentiation [6–8]. However, the effects of prostate cancer-derived exosomes on osteoclasts remain unclear.

Exosomes are extracellular vesicles with a diameter of 30–150 nm and a density of 1.13–1.19 g/ml. Whether in a physiological state or pathological state, cells can secrete exosomes to transfer certain proteins, lipids, and nucleic acids to the recipient cells by endocytosis or membrane fusion for transcellular regulation [9]. These exosomes perform various functions in immune response, antigen presentation, cell migration, cell differentiation, tumor progression, and bone metabolism [10, 11]. It has been shown that exosomes derived from melanoma cells and labeled with fluorescent dye can infiltrate lung and bone tissues, advancing development of metastases [12]. In prostate cancer, more microvesicles are present in metastatic sites than in normal tissues [13]. However, the underlying interaction has yet to be elucidated.

MicroRNAs (miRNAs) are noncoding RNAs of approximately 22 nucleotides that mainly repress gene expression at the posttranslational level by imperfect base pairing to complementary sequences in the 3′ untranslated region of mRNA [14]. It has been well established that miRNAs play an important role in various cellular processes such as tumor progression, immune regulation, and damage repair [15]. A recent study found that silencing miR-214-3p in osteoclasts significantly enhanced bone resorption and weakened the osteolytic metastasis of breast cancer [16]. Furthermore, miR-214 can enhance the bone-resorbing ability of osteoclasts and increase the expression of osteoclast markers such as Acp5, Ctsk, and Mmp9 [17]. Researchers have suggested that miR-214 can activate the PI3K/Akt pathway by targeting Pten to positively regulate osteoclastogenesis, which indicates that miR-214 is a strong contributor to osteoclast differentiation [17].

In this study, we aimed to explore the effects of prostate cancer exosomes on osteoclast differentiation and the role of miR-214 in the process. We isolated exosomes from prostate cancer cell line PC-3 and cocultured the exosomes with osteoclast precursor cells. We found that PC-3-derived exosomes remarkably inhibited differentiation of osteoclasts by downregulating miR-214 and repressing the NF-*κ*B signaling pathway. Thus, miR-214 upregulation could become a potential therapeutic method to attenuate prostate cancer bone metastasis.

## 2. Materials and Methods

### 2.1. PC-3 Cell Culture

PC-3 cells were purchased from the Chinese Academy of Sciences Type Culture Collection. PC-3 cells were cultured in Roswell Park Memorial Institute-1640 medium (RPMI-1640; Gibco, USA) supplemented with 10% fetal bovine serum (Biological Industries, Israel), 100 units/ml penicillin, and 100 mg/ml streptomycin (Gibco) at 37°C with 5% CO_2_ atmosphere. The medium was replaced every 2–3 days. When PC-3 cells reached 80% confluence, the medium was substituted with RPMI-1640 with nonexosome serum (Thermo Fisher Scientific, USA) for 2 days. Then, the cell culture supernatant was collected for exosome isolation.

### 2.2. Exosome Isolation

For exosome isolation, ultracentrifugation was applied as described previously [18]. The PC-3 cell culture supernatant mentioned in the previous section was harvested and centrifuged at 300×g and 4°C for 10 min to remove floating cells. Further centrifugation at 10,000×g and 4°C for 60 min was performed to remove cell debris. Then, the supernatant was passed through a 0.22-*μ*m filter and ultracentrifuged at 120,000×g and 4°C for 2 h using an XPN-100 rotor (Beckman Coulter, USA). The exosome pellet was rinsed with Dulbecco's phosphate-buffered saline (DPBS), and the ultracentrifugation at 120,000×g was repeated. After that, the supernatant was discarded carefully, and the exosome pellet was resuspended gently with DPBS. The exosome protein content was determined by BCA protein assay.

### 2.3. Exosome Characterization

We determined the number and size distribution of the exosomes with a NanoSight LM10 (Malvern, UK). One milliliter of sample was injected into the sample chamber with a sterile syringe. All measurement steps were conducted according to the manufacturer's guidelines. Moreover, the morphology of exosomes was observed by transmission electron microscopy (TEM). Five microliters of sample was dropped onto carbon-coated 200-mesh copper grids for 1-min incubation. Extra liquid was absorbed gently by filter paper around the border of the grids. Then, the sample was negatively stained with 2% aqueous solution of phosphotungstic acid for 30 s. Extra liquid was absorbed by filter paper again. The grids were examined using the H-7650 TEM (Hitachi, Japan) at 80 kV. After being heated for 1 min, the particle morphology was observed. In addition, nanoparticle tracking analysis was performed to assess the size distribution of PC-3 exosomes using the NanoSight LM10 (Malvern). Furthermore, the expression of exosome markers was measured by flow cytometry using an Accuri C6 flow cytometer (Becton Dickinson, USA).

### 2.4. Human Osteoclast Induction

Osteoclasts were induced from human peripheral blood mononuclear cells (PBMCs) as previously described [19]. In brief, human peripheral blood was acquired from a healthy volunteer in a centrifuge tube primed with 1000 U/ml heparin. Written informed consent was obtained before the procedure, which was approved by the Committee of Clinical Ethics of the Zhujiang Hospital. Then, the peripheral blood was diluted 1:1 with phosphate-buffered saline (PBS) and layered gently on Histopaque-1077 (Sigma-Aldrich, USA) for centrifugation (400×g, 30 min, 25°C). Next, the buffy coat was aspirated carefully and transferred into a new centrifuge tube, in which the PBMCs were washed with PBS and centrifuged twice at 250×g for 10 min. After that, PBMCs were resuspended in complete RPMI-1640 containing 30 ng/ml macrophage colony-stimulating factor (M-CSF; Sino Biological, China) and cultured in a 6-well plate at a density of 6 × 10^6^ cells/ml/well for 3 days. Nonadherent cells were then removed, and adherent cells were considered mononuclear cells. We continued to culture the mononuclear cells with complete RPMI-1640 containing 30 ng/ml M-CSF for another 3 days for cell growth. Thereafter, cells were cultured in complete RPMI-1640 (exosome-free serum) containing 30 ng/ml M-CSF and 50 ng/ml receptor activator of nuclear factor *κ*B ligand (RANKL; Sino Biological, China) with or without various concentrations of PC-3 exosomes for 10 days. Then, osteoclasts were observed by tartrate-resistant acid phosphatase (TRAP) staining. Levels of several osteoclast differentiation marker genes were measured by qRT-PCR and western blotting.

### 2.5. TRAP Staining

Osteoclasts were stained using a TRAP staining kit according to the manufacturer's instructions (Sigma Aldrich, USA). First, osteoclasts were fixed with Fixative Solution (a combination of 25 ml citrate solution, 65 ml acetone, and 8 ml of 37% formaldehyde) for 30 s at room temperature and rinsed with deionized water three times. Then, for preparation of the staining solution, 0.5 ml Fast Garnet GBC base solution and 0.5 ml sodium nitrite solution were mixed for 30 s and added into a 100-ml beaker containing 0.5 ml naphthol AS-BI phosphate solution, 2 ml acetate solution, and 1 ml tartrate solution. Next, osteoclasts were immersed in the mixed solution and incubated at 37°C for 1 h and finally rinsed with deionized water three times. The TRAP-positive cells (containing > 3 nuclei) were observed by microscopy.

### 2.6. MiRNA Mimic/Inhibitor Transfection

MiR-214 mimic and inhibitor and negative control (NC) siRNA were purchased from GenePharma Co., Ltd. (Shanghai, China). Mononuclear cells seeded in 6-well plates until 80% confluency were transfected with 50 nM miR-214 mimic, miR-214 inhibitor, or NC using RFect siRNA transfection reagent (Baidai, China) according to the manufacturer's instructions. After incubation for 48 h, cells were cultured in fresh medium and induced by M-CSF and RANKL for 10 days.

### 2.7. Total RNA Extraction

After different treatments, total RNA extraction was performed using TRIzol reagent (TaKaRa, Japan). In brief, osteoclasts were washed with PBS twice before TRIzol (1 ml/well in a 6-well plate) was added. Then, the lysis solution was moved to an Eppendorf (EP) tube and mixed with 0.2 ml chloroform, followed by centrifugation at 12,000 rpm at 4°C for 15 min. The supernatant was transferred to a new EP tube and mixed with 0.5 ml isopropanol. After centrifugation at 12,000 rpm at 4°C for 10 min, the upper aqueous phase was removed, and the RNA precipitate was washed in 75% ethanol by centrifugation again at 12,000 rpm at 4°C for 10 min. Thereafter, the upper aqueous phase was discarded, and the RNA precipitate was air-dried at room temperature. Finally, 20 *μ*l RNase-free water was added into each EP tube to dissolve the precipitate, and the concentration and quality of total RNA were measured by a spectrophotometer.

### 2.8. RT-qPCR

Reverse transcription was performed using a PrimeScript RT reagent kit (TaKaRa). QPCR analysis was conducted using a SYBR Premix Ex Taq II kit (TaKaRa) and Applied Biosystems StepOnePlus Real-Time PCR System (Thermo Fisher Scientific) according to the manufacturers' instructions. Primers were designed by Sangon Biotech Co., Ltd. (Shanghai, China): miR-214 (forward: 5′-ACACTCCAGCTGGGACAGCAGGCACAGACAG-3′, reverse: 5′-CTCAACTGGTGTCGTGGA-3′); U6 (forward: 5′-CTCGCTTCGGCAGCACA-3′, reverse: 5′-AACGCTTCACGAATTTGCGT-3′); cathepsin K (forward: 5′-TGCCCACACTTTGCTGCCGA-3′, reverse: 5′-GCAGCAGAACCTTGAGCCCCC-3′); Nfatc1 (forward: 5′-CACCGCATCACAGGGAAGAC-3′, reverse: 5′-GCACAGTCAATGACGGCTC-3′); glyceraldehyde-3-phosphate dehydrogenase (Gapdh, forward: 5′-CCGCATCTTCTTTTGCGTCG-3′, reverse: 5′-CCCAATACGACCAAATCCGTTG-3′). U6 and Gapdh were used as internal references.

### 2.9. Western Blotting

A bicinchoninic acid protein assay kit (Beyotime, China) was used for cell lysis and protein concentration measurement. Extracted protein was mixed with 1× loading buffer and boiled for 10 min before 10% sodium dodecyl sulfate-polyacrylamide gel electrophoresis. Then, protein from the gel was transferred onto polyvinylidene fluoride membranes (Boster, USA). These membranes were subsequently blocked in 3% bovine serum albumin (BSA) at room temperature for 1 h and incubated with primary rabbit monoclonal antibody (anti-CTSK, NFATc1, p65, p-p65, IKBA, p-IKBA, or GAPDH; Boster) at 4°C for 12 h. After washing with Tris-buffered saline with Tween 20 (TBST) on a rocking table three times, the membranes were incubated with goat anti-rabbit IgG secondary antibody (Boster) at room temperature for 1 h and washed with TBST three times again. The blots were observed using a Tanon 4200 SF automated fluorescence chemiluminescence image analysis system (Tanon, China).

### 2.10. Statistical Analyses

All data are presented as mean values ± standard deviations. Comparisons were performed using Student's* t*-test with GraphPad Prism version 6.0.* P* < 0.05 considered to indicate statistical significance. All experiments were repeated at least three times.

## 3. Results

### 3.1. Characteristics of PC-3 Exosomes

**Figure 1:**
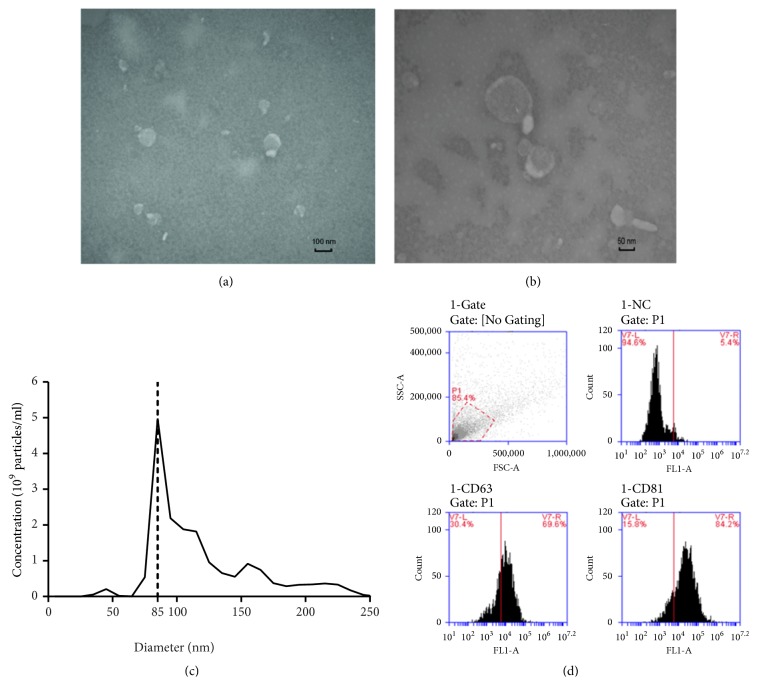
Characterization of PC-3-derived exosomes. (a) Electron microscopic images of exosomes isolated from PC-3 cells. (b) Nanoparticle tracking analysis of isolated exosomes. (c) Flow cytometric analysis of exosome surface markers CD63 and CD81.

To investigate how prostate cancer affects bone cell growth and causes bone metastases, exosomes were isolated from the prostate cancer cell line PC-3 by ultracentrifugation. Electron microscopy revealed that the vesicles were morphologically homogeneous and had a typical cup shape (Figure 1(a)). Nanoparticle tracking analysis revealed that most vesicles ranged from 70 to 120 nm in size; the distribution was around 100 nm and peaked at 85 nm (Figure 1(b)). Furthermore, flow cytometry showed that transmembrane proteins CD63 and CD81, which are specific surface markers of exosomes, were present in 69.6% and 84.2% of exosomes, respectively (Figure 1(c)).

### 3.2. PC-3-Derived Exosomes Inhibit Osteoclast Differentiation

**Figure 2:**
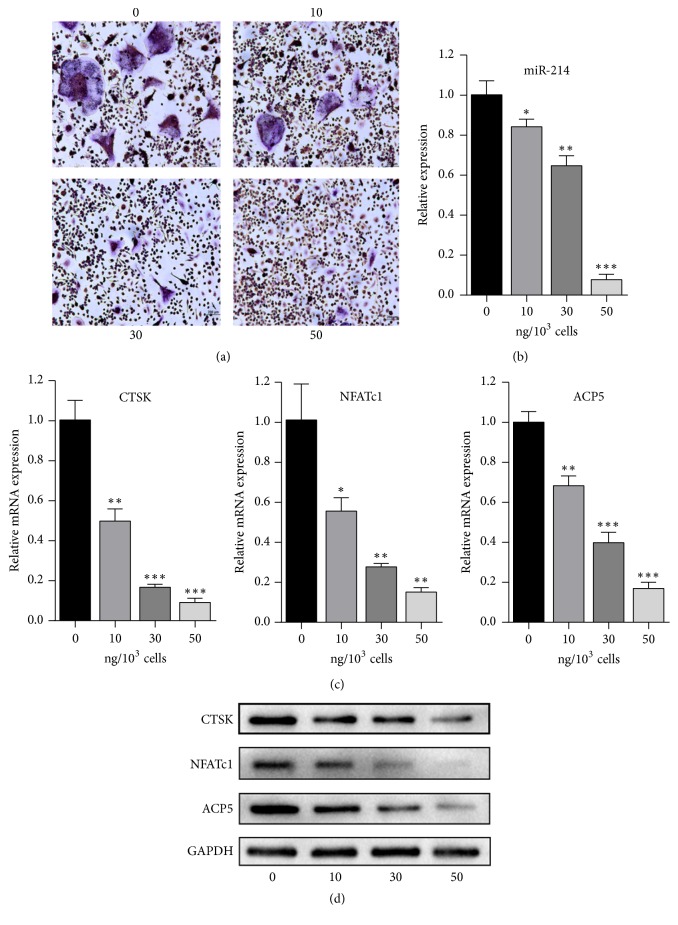
PC-3-derived exosomes inhibit osteoclast differentiation. (a) TRAP staining of osteoclasts treated with 0, 10, 30, or 50 ng exosomes/1000 cells. (b) Relative expression of miR-214 in osteoclasts treated with 0, 10, 30, or 50 ng exosomes/1000 cells. (c) qRT-PCR of CTSK, NFATc1, and ACP5 in osteoclasts after treatment with 0, 10, 30, or 50 ng exosomes/1000 cells. (d) Western blotting of CTSK, NFATc1, and ACP5 in osteoclasts after treatment with 0, 10, 30, or 50 ng exosomes/1000 cells.

To explore the effect of exosomes on osteoclasts, osteoclast precursors were cocultured with PC-3 exosomes for 10 days. Despite stimulation with M-CSF and RANKL, the differentiation of osteoclast precursors was inhibited, as shown by TRAP staining. In addition, as the concentration of exosomes increased, the inhibition became more severe (Figure 2(a)). Nearly complete inhibition of osteoclastogenesis was observed at an exosome concentration of 50 ng/1000 cells. Moreover, we detected the levels of miR-214, which were significantly reduced as the concentration of PC-3-derived exosomes increased (Figure 2(b)). At the same time, mRNA and protein expression of several specific markers of mature osteoclasts, including CTSK, NFATc1, and TRAP, was significantly decreased in the exosome groups compared with that in cells treated with only M-CSF and RANKL [20, 21] (Figures 2(c) and 2(d)). These results suggested that downregulation of miR-214 is linked to the inhibition of osteoclast differentiation.

### 3.3. MiR-214 Downregulation Inhibits Osteoclast Differentiation

**Figure 3:**
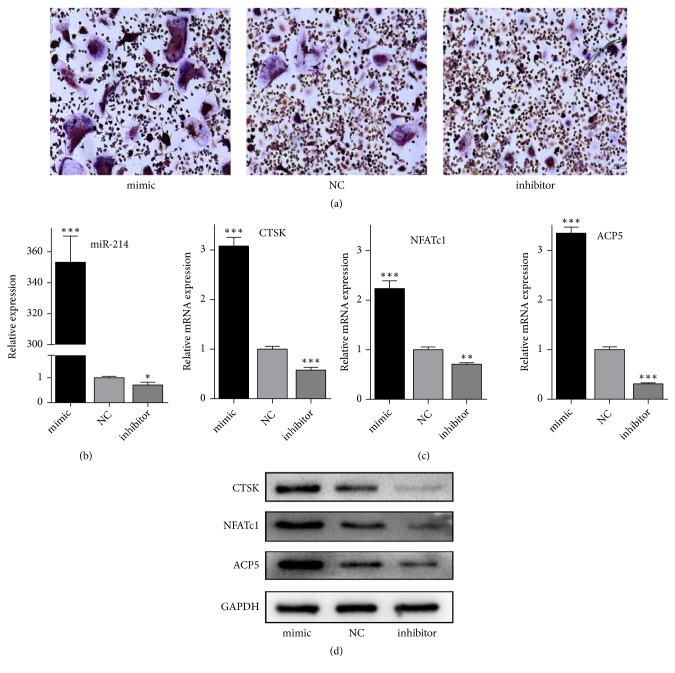
MiR-214 downregulation inhibits osteoclast differentiation. (a) TRAP staining in osteoclasts expressing miR-214 mimic or inhibitor or NC after M-CSF and RANKL induction. (b) Relative miR-214 expression in osteoclasts transfected with miR-214 mimic or inhibitor or NC was detected by qRT-PCR. (c) qRT-PCR of CTSK, NFATc1, and ACP5 in cells expressing miR-214 mimic or inhibitor or NC after M-CSF and RANKL induction. (d) Western blotting of CTSK, NFATc1, and ACP5 in cells expressing miR-214 mimic or inhibitor or NC after M-CSF and RANKL induction.

To investigate the potential effect of miR-214 on osteoclast differentiation, cells were transfected with miR-214 mimic or inhibitor or NC. TRAP staining indicated that a high level of miR-214 improved osteoclast differentiation, whereas a low level of miR-214 hampered differentiation (Figure 3(a)). Expression of miR-214 in the mimic group was 300-fold higher than that in the NC group, whereas expression in the inhibitor group was almost one-third lower than in the NC group (Figure 3(b)). The results of qPCR and western blotting were consistent; downregulated miR-214 led to decreased expression of CTSK, NFATc1, and TRAP, whereas upregulated miR-214 increased expression of these specific genes (Figures 3(c) and 3(d)). Thus, miR-214 downregulation repressed osteoclast differentiation.

### 3.4. PC-3-Derived Exosomes Block the NF-*κ*B Signaling Pathway through miR-214 Downregulation

**Figure 4:**
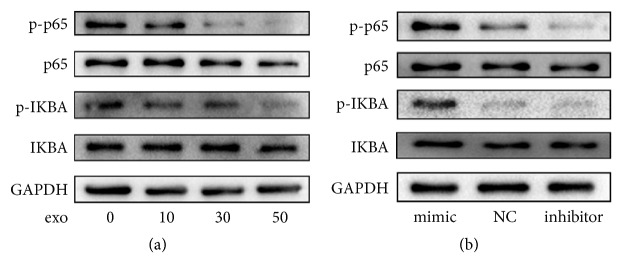
PC-3-derived exosomes block the NF-*κ*B signaling pathway through miR-214 downregulation. (a) Western blotting of p-p65, p65, p-IKBA, and IKBA in osteoclasts treated with 0, 10, 30, or 50 ng exosomes/1000 cells. (b) Western blotting of p-p65, p65, p-IKBA, and IKBA in osteoclasts transfected with miR-214 mimic or inhibitor or NC after M-CSF and RANKL induction.

For further investigation of the underlying mechanism, we focused on the response of the NF-*κ*B signaling pathway. First, we measured the protein expression of p-p65, p65, p-IKBA, and IKBA in osteoclasts cultured with various concentrations of PC-3-derived exosomes. The phosphorylation of p65 and IKBA was significantly repressed by PC-3-derived exosomes in a concentration-dependent pattern (Figure 4(a)). However, total p65 and IKBA levels were not remarkably altered. Then, we investigated whether miR-214 affected the NF-*κ*B signaling pathway. The levels of p-p65, p65, p-IKBA, and IKBA were measured in the miR-214 mimic or inhibitor and NC groups, which revealed that levels of p-p65 and p-IKBA significantly increased in the miR-214 mimic group. In contrast, their levels significantly decreased in the miR-214 inhibitor group, compared with those in the NC group. Furthermore, miR-214 overexpression and inhibition had little effect on p65 and IKBA levels (Figure 4(b)). These results strongly suggested that PC-3-derived exosomes block the NF-*κ*B signaling pathway through miR-214 downregulation.

## 4. Discussion

Our study revealed that exosomes derived from prostate cancer cell line PC-3 remarkably inhibited differentiation of osteoclasts. During this process, the expression of several marker genes of mature osteoclasts including CTSK, NFATc1, and ACP5 decreased, and miR-214 was downregulated. Furthermore, the NF-*κ*B signaling pathway was blocked. Our results are consistent with the fact that metastasis of prostate cancer is mainly osteoblastic, in which bone formation is enhanced and bone resorption is weakened [22, 23]. To further explore the mechanism underlying the interaction of PC-3 exosomes and osteoclasts, we focused on the potential role of miR-214. We found that miR-214 overexpression promoted osteoclast differentiation, whereas miR-214 downregulation repressed the differentiation. In addition, we found that a low level of miR-214 inhibited activation of the NF-*κ*B signaling pathway, which suggests the importance of miR-214 in osteoclast differentiation. Therefore, miR-214 upregulation, promoting osteoclastogenesis, may resist osteoblastic metastasis of prostate cancer.

Previously, a study reported that exosomes derived from murine prostate cancer cell line TRAMP-C1 inhibited differentiation of murine osteoclasts [24]. Researchers also showed that exosomes derived from prostate cancer cells promoted osteogenic differentiation of human mesenchymal stem cells by delivering miR-940 [25]. These results indicated that prostate cancer cells induce bone metastasis by promoting osteoblast differentiation and repressing osteoclast differentiation, so as to enhance bone formation and inhibit bone resorption. It is worth noting that a study indicated that exosomes derived from lung adenocarcinoma cells promoted osteoclast differentiation [26]. Because lung cancer cells commonly induce osteolytic bone metastasis according to clinical reports, our results are reasonable and demonstrate the diverse functions of exosomes [25].

Furthermore, our work demonstrated that PC-3 exosomes inhibit osteoclast differentiation by blocking the NF-*κ*B signaling pathway. NF-*κ*B is a transcription factor required for osteoclast differentiation and growth [27]. It plays a crucial role in the early stage of osteoclast fusion through activating c-Fos and NFATc1 [28, 29]. In the classical pathway, the phosphorylation of IKBA initiates p50/p65 dimers, which subsequently translocate to the nucleus and bind to DNA sequences, activating transcription [30]. Accordingly, cells release many proinflammatory cytokines to promote osteoclast formation [31].

In our study, PC-3 exosomes significantly decreased the level of miR-214 in osteoclasts. Further, miR-214 downregulation inhibited osteoclast differentiation through repressing the NF-*κ*B signaling pathway, which is consistent with a previous study [17]. MiR-214 is an important regulator in bone homeostasis and bone-related diseases including osteoporosis, osteosarcoma, and bone metastases [32]. In addition to promoting osteoclastogenesis, miR-214 can inhibit osteoblast differentiation by targeting ATF4 [33]. Therefore, therapeutic miR-214 mimics may attenuate the progression of prostate cancer bone metastases. However, further investigation is necessary to clarify the mechanism of miR-214 regulation.

To our knowledge, this is the first study to investigate the inhibitory effects of PC-3 exosomes on osteoclast differentiation. Nevertheless, the study still had limitations. Firstly, we failed to identify a specific molecule (RNA/protein) in exosomes that may be a main contributor to inhibition of osteoclast differentiation. Secondly, our work was carried out only in vitro, and the effect of PC-3 exosomes on animal bone remodeling warrants further research.

## 5. Conclusions

In this study, we found that exosomes derived from prostate cancer cell line PC-3 remarkably inhibited differentiation of osteoclasts by downregulating miR-214 and repressing the NF-*κ*B signaling pathway. Our findings suggest that miR-214 upregulation could become a potential therapeutic method to attenuate prostate cancer bone metastasis.

## Data Availability

The data used to support the findings of this study are included within the article.
